# The Metabolic Syndrome: Emerging Novel Insights Regarding the Relationship between the Homeostasis Model Assessment of Insulin Resistance and other Key Predictive Markers in Young Adults of Western Algeria

**DOI:** 10.3390/nu12030727

**Published:** 2020-03-10

**Authors:** Mohammed Ilyes Belhayara, Zoheir Mellouk, Mohammed Seddik Hamdaoui, Malika Bachaoui, Omar Kheroua, Willy J. Malaisse

**Affiliations:** 1 Laboratory of Physiology of Nutrition and Food Safety, Department of Biology, Faculty of Natural and Life Sciences, Université Oran1, 31000 Oran, Algeria; i.belhayara@netcourrier.com (M.I.B.); z.mellouk@netcourrier.com (Z.M.); omar_kheroua@hotmail.com (O.K.); 2 Department of internal medicine, University Hospital Institution of Oran, 31000 Oran, Algeria; seddik8142@outlook.com (M.S.H.); mbachaoui@yahoo.fr (M.B.); 3 Department of Biochemistry, Université Libre de Bruxelles, B-1070 Brussels, Belgium

**Keywords:** metabolic syndrome, insulin resistance, young adults, obesity, biomarkers, adipokines, insulinogenic index, HOMA-IR, GLP-1

## Abstract

Several biological markers have been identified as risk factors for cardiovascular disease and are associated with increased risk of metabolic syndrome (MetS). This study provides a factual information on promising biomarkers that are associated with MetS and can aid in early detection and management of MetS in young adults of Western Algeria. We studied a total of one hundred subjects aged between thirty and forty years with MetS, in which anthropometric measurements, insulin resistance, C peptide and HbA1c, lipid profile, circulating adipokines and glucagon-like peptide-1 were measured by suitable methods, in comparison to two groups of control. MetS is closely linked to altered glucose homeostasis, the plasma insulin/glucose ratio; i.e., the insulinogenic index helps to estimate the level of insulin secretion and also for assessing β-cell function. The correlation between homeostasis model assessment insulin resistance index (HOMA-IR) and HbA1c, body mass index or plasma triglycerides yielded positive and significant values. Biomarkers with a known and predictable association with MetS can provide a means to detect those at risk and intervene as needed. This could significantly decrease the burden complications impose on patients and the healthcare system.

## 1. Introduction

The transition to modernity that accompanied the globalization is often associated with changes in food habits. This contributed to an upsurge in the frequency of nutrition-related diseases, including both under- and over-nutrition. Indeed, regardless of region and countries, diet is rapidly passing from a traditional to a westernized pattern [1,2,3]. Over the last few years, the three countries of North Africa: Algeria, Tunisia and Morocco are encountering an active epidemiological and nutritional transition. This has resulted in an increase in the incidence of some metabolic disorders as overweight and obesity and in the prevalence of comorbidities such as type 2 diabetes mellitus (T2DM) and cardiovascular diseases (CVDs) [4,5,6].

Obesity is a natural consequence of over nutrition and sedentary lifestyle. In 2016, more than 1.9 billion adults were overweight and, from these, more than 650 million were obese. If recent trends continue unabated, up to 20% of the world’s adult population (1.2 billion individuals) is expected to be obese by 2030 [7,8]. Persistent obesity dysregulates metabolic processes, including action of insulin on glucose-lipid-free fatty acid metabolism, and severely affects processes controlling blood glucose, blood pressure and lipids. Thus begins a cluster of conditions: dysglycemia, dyslipidemia, hypertension, proinflammatory state and procoagulant state, known as the metabolic syndrome (MetS) [9]. This new public health burden is widely studied and poses a major challenge for the coming years. Approximately 20–30% of the adult population in most countries is affected by MetS. The prevalence is dependent on age, gender, race and diagnostic criteria. The incidence of MetS is expected to increase to approximately 53% at 2035 [10].

Although the exact etiology of MetS has not yet been completely elucidated, many cross-sectional or longitudinal studies have shown that MetS is strongly associated with insulin resistance, inflammation, endothelial dysfunction and risk of CVDs [11]. Therefore, some research groups use a mixture of several biological markers as risk factors for MetS. Many of these biomarkers are interrelated in how they play a role in MetS, so correlations between biomarkers would be helpful to assess patients. With this early detection, early intervention is also possible and could be an effective means to diminish the widespread effects this syndrome. Biomarkers could also provide a mechanism to personalize treatment given the etiology differences amongst individuals [12,13].

Traditionally, assessment of MetS and risk for CVDs has involved the analysis of plasma lipids, including total cholesterol (TC), triglycerides (TGs), high-density lipoprotein cholesterol (HDL-C), low-density lipoprotein cholesterol (LDL-C), insulin and C-peptide levels. An early intervention to normalize circulating lipids has been shown to reduce cardiovascular complications and mortality [14]. Biomarkers linking adiposity and inflammation, such as interleukin-6 (IL-6), tumor necrosis factor α (TNF-α) and high-sensitivity C-reactive protein (hs-CRP) represent prototypic and predictive inflammatory markers of CVDs [15,16,17]. Similarly, adipocyte-derived hormones, leptin and adiponectin are emerging biomarkers of insulin resistance [18,19]. Additionally to the traditional markers, homeostasis model assessment insulin resistance index [20] (HOMA-IR; as a marker of insulin resistance), insulinogenic index (II) as a reasonable surrogate and valid marker of insulin sensitivity disturbances and beta cell function in type 2 diabetes and other cardiometabolic diseases [21], glycated hemoglobin [22] (HbA1c; as a marker for long-term glycemic control) and glucagon-like peptide-1 [23,24] (GLP-1; as a marker of insulin secretion glucose-induced), are assessed.

In a recent article, it was duly underlined that the MetS becomes increasingly obvious from an early age in Western Algeria, which experienced since a few years an important demographic and epidemiological transition characterized by drastic changes in lifestyle, rapid urbanization and environmental deterioration [25]. Clinically, the aforementioned biomarkers are all readily measured and alterations in these biomarkers may predict development of MetS. These markers are usually used as part of a medical health check-up program in Algeria. Indeed, such biomarkers identifying individuals with an aggregation of risk factors for MetS would not only serve as diagnostic criteria but help guide clinical management.

The major aim of the present report is to provide factual information on the various biomarkers recorded in two groups of control subjects and one-hundred patients affected by the MetS considered in the above-mentioned recent article, on one hand, and to document the interrelationships between selected variables, on the other hand. Both, however, may help understanding the MetS, profiling patients and probe the existence of subphenotypes. These data could provide early information in subjects prone to develop MetS.

## 2. Subjects, Materials and Methods

### 2.1. Study Location

The study was conducted in the city of Oran, an urban area located in the northwest region of Algeria; it is the second largest city in Algeria and one of the most important in Northern Africa. According to the 2018 National Census in Oran, approximately 1.98 million individuals lived in Oran. The North-Western Algeria had a population of 8.61 million. 

### 2.2. Study Population

The population under study consisted of a cohort of 100 patients (60 men and 40 women) aged between 30 and 40 years among the 2365 cases of diagnosed MetS patients that have been admitted for consultation in diabetology at the Department of Internal Medicine of both the Military Regional University Hospital of Oran (HMRUO) and University Hospital Establishment (EHU) of Oran. The criteria for the diagnostic of MetS were those defined by the National Cholesterol Education Program-Adult Treatment Panel III (NCEP-ATPIII) [26]. A subject was considered to present a MetS if 3 of the 5 following criteria were found: abdominal obesity (WC >102 cm in men and >88 cm in women); plasma TGs >1.50 g/ L; HDL-C <0.4 g/L in men and <0.5 g/L in women or under treatment of anti-lipid agents (fibrates and nicotinic acid); blood pressure >130/85 mm Hg or fasting plasma glucose >1.1 g/L (threshold decreased to 1.0 g/L in 2005 following the recommendation of the American Diabetes Association) or drug treatment for hypertension or type 2 diabetes, respectively. The exclusion criteria included: nonconsenting young adult patients or not meeting at least 3 of the 5 outreach NCEP-ATPIII criteria; type 1 diabetic patients; subjects or history of any chronic disease, such as kidney or liver diseases, and any endocrine problems, especially Cushing’s disease and thyroid dysfunctions. Pregnant women under estro-progestins drugs were excluded. The same applies to patients under weight control, corticoids, antidepressants and nonsteroidal anti-inflammatory drugs being excluded. Diabetic patients were treated with metformin (Glucophage ^®,^ Merck serono S.A.S, Darmstadt, Germany) 850 mg 2 to 3 times a day. Drug doses were stable throughout the study. No diabetic patient was submitted to insulin therapy. Oral antidiabetics, metformin in particular, and lipid lowering drugs that may interfere with adipocytokine gene expression, were stopped 3 days before blood sample collection. The study was conducted from November 2015 to December 2017.

The rest of the cohort is formed by healthy control participants selected from various administrative departments of the faculty of natural and life sciences of the University of Oran 1. They were enrolled in the study at the same period of patients’ recruitment and underwent a detailed medical and surgical history and a complete physical examination performed by cardiologist and internist, including vital signs, electrocardiogram, hematology, blood chemistry, serology and urinalysis. Inclusion criteria included: to be categorized as normal weight (BMI 18.5–24.9 kg / m2) or obese (BMI >30 kg/m^2^) and aged between 30 and 40. An individual was considered “metabolically healthy” if the following criteria were met: fasting plasma glucose <1.1 g/L without use of anti-diabetic drugs, HDL-C >0.4g/L for males and > 0.5g/L for females, TGs <1.5 g/L and LDL-C <1.6 g/L without use of lipid-lowering drugs. Exclusion criteria included: any history of chronic diseases; autoimmune and infectious diseases; current alcohol or drug abuse and smoking; more than 3 kg weight change in the past two months; recent surgery; dieting or under a controlled dietary regimen over the previous six months; receiving some type of drug therapy or complementary and alternative medicines that lowered the body weight, lipid profile or blood glucose and women could not be pregnant or lactating. These subjects’ “metabolic health” have been distributed as follows: normal-weight control subjects (BMI 18.5–24.9 kg/m^2^) and obese control subjects (BMI >30 kg/m^2^). The sex ratio was 1 for the two groups (20 male and 20 female).

### 2.3. Blood Pressure Measurement and Hypertension Definition

Blood pressure (BP) was measured on the dominant arm using an Omron 705 CP automated BP monitor (Omron Healthcare, Osaka, Japan) with subjects seated, following a 10–15 min rest; two readings were taken 5 min apart, and the mean of the two was taken as the BP when smoking and drink containing alcohol and caffeine was avoided. The measured BP was classified as optimal (SBP <120 and DBP <80 mm Hg), normal (SBP 120–129 and/or DBP 80–84 mm Hg), high-normal (SBP 130–139 and/or DBP 85–89 mm Hg) or hypertension (SBP/DBP ≥140/90 mm Hg and/or the use of antihypertensive medications with self-reported hypertension).

### 2.4. Anthropometric Measurements

All measurements were obtained in the morning time after overnight fasting status according to protocols and recorded. Body mass was recorded to the nearest 0.1 Kg on a calibrated digital scale, with subjects wearing only light clothing and without shoes. Height was measured to the closest 0.1 cm with stadiometer in standing position. Both height and weight were used to calculate body mass index (BMI) as kg/m^2^. The criterion to define obesity was based on the Quetelet Index or BMI, five types being defined: i.e., normal weight (between 18.5 and 24.9), overweight (between 25.0 and 29.9), obesity (between 30.0 and 34.9, class I), massive obesity (between 35.0 and 39.9, class II) and morbid obesity (above 40.0, class III). Waist circumference (WC) was measured by using a nonflexible tape that was placed directly on the skin with the subject facing forward, feet together, with both arms hanging freely. All WC measurements were done to the nearest 0.1 cm immediately above the iliac crest and repeated three times for a precise average measurement. 

### 2.5. Blood Sampling

Blood samples were collected after 12 h of fasting from a vein in the antecubital fossa without venous occlusion. All collections were made between 8:00 and 9:00 a.m. Whole blood specimens were collected in different tubes to obtain plasma. The samples were separated in aliquots and frozen immediately at −80 °C until biochemical analyses could be performed.

### 2.6. Plasma Lipids and Glucose

Plasma TC, HDL-C, TG, glucose and HbA1c were all measured by multiparametric automated procedure using Cobas 6000 analyzer with Roche Diagnostic’s reactives. Plasma LDL-cholesterol (LDL-C) was estimated by the Friedewald equation [27]. None of the participants had plasma TG>4 g/L, which can affect the calculation of LDL-C by the Friedwald equation.

### 2.7. Plasma Insulin, C-Peptide, Homeostasis Model Assessment of Insulin Resistance and the Insulinogenic Index Calculations

Insulinemia and C-peptide were determined by electrochemiluminescence (ECLA) assay in a random access analyzer (Cobas E411, Roche Diagnostics, Les Pennes-Mirabeau, Bouches-du-Rhône, France). The homeostasis model assessment of insulin resistance (HOMA-IR) score was calculated using the formula proposed by Matthews et al. (1985): HOMA IR = [fasting insulin (mU/mL) × fasting glucose (mmol/L)]/22.5 [28].

The insulinogenic index (II) a valid marker of beta cell function in different metabolic categories that measure the insulin response to glucose challenge was calculated from the ratio of fasting insulin concentration in microunits per milliliter and fasting glucose concentration expressed in millimolar.

### 2.8. Plasma Adipokines and Other Markers

Antigenic immunoassay procedure based on enzyme linked immunosorbent assays from RD Systems (Wiesbaden-Nordenstadt, Germany) was used for the quantification of leptin (human leptin; Quantikine); adiponectin (human adiponectin, Acrp30; Quantikine); IL-6 (human IL-6; Quantikine) and TNF-a (human TNF-a; Quantikine) carried out in a microtiter plate analyzer (human-reader; Wiesbaden, Germany). Plasma GLP-1 and hs-CRP were measured using Abcam kit (Cat N # 184857) and Cell Biolabs Assay (Cat N # STA-392), respectively.

### 2.9. Ethical Consideration

Ethical clearance was obtained from the Institutional Review Board of the University Hospital Institution of Oran (November 02, 2015, approval number ETAP-C.R.S 6) in accordance with the tenets of the Declaration of Helsinki (http://www.wma.net). The researcher explained the study to each person and gave them thorough information about the study and its purpose. In addition, we notified them of their right to terminate their participation in the study at any time without incurring any penalty. The concerned subjects having given a signed consent after having been duly informed. On occasion, the consent was orally given after explaining the aim of the biological assays. For each subject, a file was prepared to include all relevant information (age, sex, residence, ethnicity, profession, instruction, matrimonial status, family antecedents of diabetes, obesity, hypertension and vascular accident, tobacco use, physical and paraclinical data).

### 2.10. Data management and Statistical Analysis

Statistical assessment was conducted with GraphPAD Prism V.7 (GraphPAD Software, San Diego, CA, USA). Abnormal values (outliers) were excluded. Results were expressed as mean ± SEM, with a 95% confidence interval (95% CI). The mean values of the groups were compared using a Student’s unpaired *t*-test. Correlations between the homeostasis model assessment of insulin resistance and other measured variables were evaluated using Pearson’s correlation coefficient followed by regression analysis. Statistical significance was set at *p* < 0.05.

## 3. Results

### 3.1. Glycemia as a First Marker of the Metabolic Syndrome in Young Adults of Western Algeria

More than 2000 distinct data were taken into consideration in our recent study concerning the increasing occurrence of the MetS in young adults living in Western Algeria [24]. Based on such primary information as age, sex, body weight and height, glycemia and insulinemia, the comparison between fifteen markers of the MetS in control subjects and either male or female patients affected by the MetS, non-obese overweight and obese subjects, as well as non-diabetic versus diabetic patients, was achieved.

Table 1 provides the mean values (± SEM) for fifteen variables measured in twenty control subjects and in either twenty-five non-diabetic or seventy-five diabetic patients affected by the metabolic syndrome. The glycemia averaged 4.78 ± 0.11 (*n* = 20), 6.02 ± 0.08 (*n* = 25) and 8.00 ± 0.05 (*n* = 75) in the control subjects, non-diabetic and diabetic patients, respectively.

The results of the correlation analyses between glycemia and selected variables in the non-diabetic and diabetic patients affected by the MetS are listed in Table 2. In the non-diabetic patients, significant positive correlations were found between glycemia and either HOMA-IR index (*p* < 0.01), C-peptide (*p* < 0.01) or HbA1C (*p* < 0.01). In the same patients, significant negative correlations were observed between glycemia and either TNF-α (*p* < 0.01) or LDL-C (*p* < 0.05).

In the larger group of diabetic patients, highly significant correlations prevailed between glycemia and BMI, insulinemia, HOMA-IR index, C-peptide, HbA1C, HDL-C, TGs/HDL-C ratio, adiponectin, IL-6 and TNF-α (*p* < 0.01 in all cases), whilst significant correlations with a higher p-value (*p* < 0.05) were observed between glycemia and either total cholesterol or GLP-1. These correlations provided a negative Pearson r in the case of HDL-C, adiponectin, TNF-α and GLP-1. Even in the diabetic patients, no significant correlation was detected between glycemia and LDL-C, leptin or hs-CRP.

In addition to the eventual presence of hyperglycemia, the most obvious difference between the non-diabetic and diabetic patients concerned the higher HOMA-IR index and lower TNF-α value recorded in the diabetic patients than in the non-diabetic one. No significant difference between non-diabetic and diabetic patients was observed for HDL-C, TGs, leptin, adiponectin, IL-6, hs-CRP and GLP-1. 

It should be stressed that the presence of a significant correlation between two distinct variables does not necessarily imply that these variables are directly interconnected by a cause-to-effect link. Nevertheless, the present findings are compatible with the view that the imbalance between food intake and energy expenditure leading to an increase in BMI (kg/m^2^) from 22.1 ± 0.3 in non-obese control subjects to 31.6 ± 0.4 in the non-diabetic MetS patients and 33.1 ± 0.3 in the diabetic MetS patients participates in the parallel increase of glycemia from 4.78 ± 0.11 in the control group to 6.02 ± 0.08 and 8.00 ± 0.09 in the non- diabetic and diabetic MetS patients.

### 3.2. Relevance of the Insulinogenic Index to the Insulin Secretory Response of the Endocrine Pancreas to Glucose in the Metabolic Syndrome

The plasma insulin/glucose ratio, i.e., the insulinogenic index (II) is not listed among the biochemical markers of the MetS. Yet, extensive prior studies conducted in several animal models of non-insulin-dependent diabetes have validated the use of this index to assess the pancreatic islet β-cell responsiveness to glucose [28,29,30,31,32]. The aim of the present study is to investigate in one-hundred young adults affected by the MetS the possible relevance of this index to the pathogenesis of the MetS.

Table 3 provides the mean found in non-obese and obese control subjects, as well as male and female MetS patients, non-diabetic and diabetic MetS patients and overweight and obese MetS patients. As expected, such a ratio was about twice higher (*p* < 0.01) in obese than non-obese control subjects. Despite comparable values for the BMI in the 60 male MetS patients (32.67 ± 0.30 kg/m^2^) and 40 female MetS patients (32.75 ± 0.45 kg/m^2^), the plasma insulin/glucose ratio was, modestly but significantly (*p* < 0.01), higher in female than male patients. The overall mean value for the plasma insulin/glucose ratio in the 100 MetS patients averaged 4.31 ± 0.07 μU/mol and, as such, was significantly lower in these patients than in the obese control subjects. The II failed, however, to differ significantly (*p* = 0.70) in overweight and truly obese MetS patients. The BMI averaged 31.57 ± 0.40 kg/m^2^ and 33.30 ± 0.22 kg/m^2^, respectively, in 25 non-diabetic and 75 diabetic MetS patients. Despite such a minor difference in BMI, the mean II was obviously significantly higher (*p* < 0.01) in the non-diabetic than in the diabetic patients. 

In the non-diabetic patients, only the correlations between the II and either HbA1C (*p* < 0.01) or TNF-α (*p* < 0.05) provided significant positive values, whilst that concerning GLP-1 provided a significant (*p* < 0.01) negative value. In the diabetic patients, however, significant positive correlations were observed between the II and either BMI (*p* < 0.01), C-peptide (*p* < 0.01), HOMA-IR index (*p* < 0.01), leptin (*p* < 0.01), hs-CRP (*p* < 0.01), TC (*p* < 0.05), TG (*p* < 0.05) or IL-6 (*p* < 0.05), whilst the correlation between II and GLP-1 reached a significant (*p* < 0.01) negative value (Table 4).

### 3.3. Correlation between the Homeostasis Model Assessment and Twelve other Selected Variables of the Metabolic Syndrome

As already alluded to, the BMI (kg/m^2^), glycemia (mM), insulinemia (μU/mL), plasma C-peptide concentration (ng/mL), HOMA-IR (mM.μU/mL), HbA1C (percent), TC (g/L), HDL-C (g/L), LDL-C (g/L), TGs (g/L), leptin (ng/mL), adiponectin (μg/mL), IL-6 (pg/mL), TNF-α (pg/mL), hs-CRP (ng/L) and GLP- 1 (pmol/L) were measured in 60 male and 40 female subjects aged between 30 and 40 years and affected by the MetS. Correlation analyses between the HOMA-IR index and 12 selected variables were examined in either male or female patients. The HOMA-IR averaged 236.4 ± 7.7 and 248.03 ± 8.6 in the 60 males and 40 females MetS patients, respectively, as compared to 53.5 ± 5.0 in 20 control subjects.

Table 5 provides the correlation coefficient and statistical probability of these analyses ranged in order of either decreasing statistical significance (positive correlation coefficients) or increasing statistical significance (negative correlation coefficients).

The highest values for the correlation index concerned the comparison between HOMA-IR and HbA1C.

The BMI averaged 22.67 ± 0.34 (*n* = 60) and 32.75 ± 0.45 (*n* = 40) in male and female MetS patients, respectively, as distinct from 22.10 ± 0.31 in control subjects (*n* = 20). The correlation analysis between HOMA-IR index and BMI yielded correlation coefficients of + 0.7077 and + 0.7045 (Table 4), line 4 (*p* < 0.01 in both cases) in male and female MetS patients, respectively. 

The correlation between HOMA-IR and TG also achieved statistical significance (*p* < 0.01) in both male and female MetS patients. 

No other significant correlation (*p* < 0.05) between HOMA-IR index and another selected variable was obtained in both male and female MetS patients. The most obvious lacks of significant correlation with the HOMA-IR index in both male and female MetS patients concerned TC and LDL-C.

## 4. Discussion

MetS is a progressive condition that encompasses a wide array of disorders with specific metabolic abnormalities presenting at different times. These abnormalities can be detected and monitored via plasma biomarkers [11,12]. This study conducted to compare multiple circulating biomarkers and their association with MetS among young adults in Western Algeria and to determine which biomarkers are more strongly related to MetS. We selected relatively inexpensive markers, which could be widely used in clinical practice in our community medical health check-up program, rather than markers typically available for research purposes only.

Glycemia was the first marker of the MetS in our population study. In terms of a cause-to-effect link emphasis is placed on the imbalance between food intake and energy expenditure and resulting increase in glycemia. These findings stress the risk of obesity at early ages, with alterations in some of the components of MetS. Based on our own work, it also appears self-evident that we need to intervene earlier in the glucose trajectory, to maintain glucose homeostasis as close to “normal” as possible and before people are given a biochemical diagnosis of diabetes. Blood glucose is a continuum of risk for CVDs, and it is likely that changes in the vascular epithelium that cannot be reversed simply by lowering blood glucose have already occurred by the time of diabetes diagnosis. [33,34]. More research, basic, translational and clinical, is needed to better understand interaction of dietary and lifestyle factors in the genesis of diabetes and other metabolic diseases in humans. This knowledge can help us better reinforce selection in “quality” food consumption.

The II, i.e., the plasma insulin/glucose ratio, is not listed among the markers of the MetS. Yet, the present findings reinforce the usefulness of this II in assessing the changes occurring in patients affected by the MetS the insulin secretory response of the endocrine pancreas to extracellular glucose considered as the major regulator of insulin secretion. [35,36] Thus, the present findings are compatible with the well-known higher rate of insulin release in either obese control subjects or non-diabetic MetS patients than in non-obese control subjects. They also suggest that the insulin secretory response of pancreatic islet B cells to glucose is modestly but significantly decreased in MetS as compared to obese control subjects [37,38,39,40]. Such a difference was observed when comparing either male or female MetS patients and either overweight or obese MetS patients, as well as diabetic MetS patients to obese control subjects. It was not observed, however, when comparing non-diabetic MetS patients to obese control subjects. The obvious difference between the mean values for the II found in non-diabetic versus diabetic MetS patients appears compatible with the hypothesis that, in patients affected by the MetS, the insulin secretory response of the endocrine pancreas to extracellular glucose is indeed perturbed in parallel with the worsening of glucose homeostasis [37,41,42,43,44]. Although the II is not corrected for the threshold value of glycemia required to augment insulin release above the basal value, the present finding of a much higher plasma insulin/glucose ratio in obese than non-obese control subjects is consistent with the hypothesis that obesity, as caused by an imbalance between food intake and energy expenditure, eventually leads to a rise in insulin output and, possibly, even in the insulin secretory responsiveness of the endocrine pancreas to a given hexose concentration [42,45]. It should not be ignored, however, that the rate of insulin secretion by the endocrine pancreas at a given extracellular glucose concentration may also be modulated by the amount of insulin stored in the pancreatic gland. In other words, a decrease of the II, as observed for instance in diabetic MetS patients, does not inform on the question whether the impaired release of insulin is attributable to an alteration of the process of glucose recognition by the B cells as an insulinotropic agent, a decreased availability of insulin in the pancreatic gland or a combination of the latter two processes [35,46,47]. The fact that a lower II in MetS patients than in control obese subjects prevailed in diabetic MetS patients, but not so in non-diabetic MetS patients, apparently argues in favor of the second hypothesis mentioned in the preceding sentence, i.e., a decrease of the pancreatic insulin stores otherwise available to ensure a suitable release of insulin. Alternatively, however, the dramatic difference between the II in non-diabetic and diabetic MetS patients may be attributable to the phenomenon of so-called glucotoxicity caused by the accumulation of glycogen in the pancreatic islet B cells [48,49,50]. Prior investigations have documented that such an accumulation of glycogen accounts in diabetic subjects for both the early and transient paradoxical inhibition of insulin release and anomeric perturbation of glucose-induced insulin release both observed after intravenous administration of the hexose [51,52]. In conclusion, therefore, it is proposed that relevant information could be reached by following in the same MetS patients and, over a suitable period, the progressive changes in the early insulinemic response to either α- or β-D-glucose or the hexose at anomeric equilibrium following their intravenous administration.

The HOMA-IR model is used to yield an estimate of insulin sensitivity and B-cell function from fasting plasma insulin and glucose concentrations. The present findings demonstrate that HbA1c, BMI and plasma TG are the sole among 12 variables to achieve, both in male and female MetS patients, a highly significant correlation with the HOMA-IR index is in fair agreement with some prior observations [14,53,54,55]. In our opinion, this finding provides further support to the view that an altered regulation of plasma D-glucose and/or insulin concentration(s), as assessed by the HOMA-IR index, plays a key role in the non-enzymatic glycation of hemoglobin and regulation of plasma triglyceride concentration and coincides with the increase in BMI. These findings are in concordance with many previous studies [30,56,57,58,59,60]. The perturbation of glucose homeostasis, as judged by the coefficient of glucose assimilation during an intravenous glucose tolerance test, in obese subjects was already documented more than 55 years ago in a lecture given at the Académie Royale de Médecine de Belgique [61]. In other words, the following sequence of events could be proposed as a far from uncommon, even if not always operative, process involved in the development of the MetS, leading from obesity, as resulting for instance from excess food intake, to glucose intolerance, hyperglycemia, non-enzymatic glycation of hemoglobin and hypertriglyceridemia. It should not be ignored, however, that, even in non-insulin-dependent diabetes mellitus, other inherited or acquired priming metabolic or hormonal perturbation may also eventually lead to the occurrence of the MetS. Nevertheless, the key roles ascribed to obesity, altered glucose homeostasis and insulin resistance in the development of the MetS will be further emphasized in further reports dealing with the same one -hundred young MetS patients as those considered in the present study but centered on the relevance of alteration in BMI in the pathogenesis of the MetS.

## 5. Conclusions

MetS is closely linked to altered glucose homeostasis, and measuring II has several advantages: it involves fewer complex protocols, needs less costs and utilizes a physiological route of glucose administration. It is proposed that a decrease in the pancreatic insulin stores otherwise available to ensure a suitable release of insulin may play a role in the perturbation of the II. The latter perturbation may also involve a phenomenon of so-called glucotoxicity, as could be assessed in patients affected by the MetS by occurrence or either a paradoxical early decrease in insulinemia and/or an altered anomeric specificity of the pancreatic insulin release after intravenous administration of glucose. The HOMA-IR index provides further support to the view that an altered regulation of plasma glucose and/or insulin plays a key role in the non-enzymatic glycation of hemoglobin and control of plasma TGs concentration and coincides with an increase in BMI. Further research is encouraged to determine the efficacy of applying these biomarkers to diagnosis and treatment in a clinical setting.

## Figures and Tables

**Table 1:** Biochemical parameters in control group, diabetic and non-diabetic metabolic syndrome (MetS) patients.

Parameter	Control Group	Non-Diabetic Group	Diabetic Group
Glycemia (mM)	4.78 ± 0.11 (*n* = 20)	6.02 ± 0.08 (*n* = 25) ^a*^	8.00 ± 0.09 (*n* = 75) ^b*,c*^
Insulinemia (µU/mL)	11.05 ± 0.88 (*n* = 20)	30.38 ± 0.37 (*n* = 25) ^a*^	32.33 ± 0.45 (*n* = 75) ^b*,c#^
C-peptide (ng/mL)	1.44 ± 0.06 (*n* = 20)	2.34 ± 0.07 (*n* = 25) ^a*^	2.94 ± 0.06 (*n* = 75) ^b*,c*^
HOMA-IR (mM µU	53.5 ± 4.95 (*n* = 20)	183.47 ± 4.05 (*n* = 25) ^a*^	260.22 ± 5.94 (*n* = 75) ^b*,c*^
HbA1C (%)	5.29 ± 0.05 (*n* = 20)	5.92 ± 0.05 (*n* = 25) ^a*^	6.80 ± 0.06 (*n* = 75) ^b*,c*^
Total cholesterol (g/L)	1.60 ± 0.04 (*n* = 20)	2.09 ± 0.01 (*n* = 25) ^a*^	2.03 ± 0.01 (*n* = 75) ^b*,c#^
HDL-C (g/l)	0.57 ± 0.01 (*n* = 20)	0.41 ± 0.01 (*n* = 25) ^a*^	0.43 ± 0.01 (*n* = 75) ^b*^
LDL-C (g/L)	1.00 ± 0.04 (*n* = 20)	1.26 ± 0.01 (*n* = 25) ^a*^	1.20 ± 0.01 (*n* = 75) ^b*,c#^
Triglycerides (g/L)	1.06 ± 0.05 (*n* = 20)	1.99 ± 0.03 (*n* = 25) ^a*^	1.99 ± 0.04 (*n* = 75) ^b*^
Leptin (ng/mL)	7.11 ± 1.17 (*n* = 12)	17.81 ± 1.58 (*n* = 11) ^a*^	17.99 ± 0.87 (*n* = 55) ^b*^
Adiponectin (µg)	7.43 ± 0.69 (*n* = 12)	3.92 ± 0.31 (*n* = 09) ^a*^	3.91 ± 0.17 (*n* = 53) ^b*^
Interleukin-6 (pg/mL)	7.70 ± 0.73 (*n* = 14)	12.57 ± 0.97 (*n* = 12) ^a*^	12.99 ± 0.61 (*n* = 51) ^b*^
TNF-α (pg)	14.90 ± 2.27 (*n* = 14)	25.31 ± 2.07 (*n* = 11) ^a*^	19.67 ± 1.22 (*n* = 55) ^b*,c#^
Hs-CRP (mg/L)	1.52 ± 0.10 (*n* = 20)	4.80 ± 0.67 (*n* = 10) ^a*^	5.23 ± 0.33 (*n* = 40) ^b*^
GLP-1 (pmol/L)	24.52 ± 0.55 (*n* = 20)	13.09 ± 0.72 (*n* = 14) ^a*^	12.64 ± 0.55 (*n* = 48) ^b*^

^a^ Non-diabetic versus control, ^b^ diabetic versus control and ^c^ diabetic versus non-diabetic. * *p* < 0.01 and ^#^ *p* < 0.05. HOMA-IR: homeostasis model assessment insulin resistance index.

**Table 2:** Correlation analyses between glycemia and selected variables in non-diabetic and diabetic MetS patients.

**BMI**		**Insulinemia**		**HOMA-IR Index**	
**Non-diabetic**	**Diabetic**	**Non-diabetic**	**Diabetic**	**Non-diabetic**	**Diabetic**
+0.2849 (*p* = 0.16)	+0.5558 (*p* < 0.01)	+0.0914 (*p* = 0.42)	+0.5456 (*p* <0.01)	+0.8281 (*p* < 0.01)	+0.8643 (*p* < 0.01)
**C-Peptide**		**Glycated hemoglobin (HbA1C)**		**Total cholesterol**	
**Non-diabetic**	**Diabetic**	**Non-diabetic**	**Diabetic**	**Non-diabetic**	**Diabetic**
+0.6314 (*p* < 0.01)	+0.6270 (*p* < 0.01)	+0.6700 (*p* < 0.01)	+0.6857 (*p* < 0.01)	−0.3422 (*p* = 0.09)	+0.2304 (*p* < 0.05)
**HDL-cholesterol**		**LDL-cholesterol**		**Triglycerides to HDL-C ratio**	
**Non-diabetic**	**Diabetic**	**Non-diabetic**	**Diabetic**	**Non-diabetic**	**Diabetic**
+0.2483 (*p* = 0.23)	−0.2930 (*p* < 0.01)	−0.4257 (*p* < 0.05)	+0.1147 (*p* = 0.32)	+0.2928 (*p* = 0.15)^b^	+0.4309 (*p* < 0.01)^a^
**Leptin**		**Adiponectin**		**Interleukin-6**	
**Non-diabetic**	**Diabetic**	**Non-diabetic**	**Diabetic**	**Non-diabetic**	**Diabetic**
−0.3901 (*p* = 0.21)^a^	−0.0138 (*p* = 0.92)^b^	−0.1971 (*p* = 0.56)^c^	−0.3639 (*p* < 0.01)^d^	−0.1398 (*p* = 0.66)^a^	+0.3519 (*p* < 0.01)^d^
**TNF-α**		**hs-CRP**		**GLP-1**	
**Non-diabetic**	**Diabetic**	**Non-diabetic**	**Diabetic**	**Non-diabetic**	**Diabetic**
−0.7927 (*p* < 0.01)^e^	−0.3393 (*p* < 0.01)^f^	-0.3317 (*p* = 0.31)^c^	+0.1841 (*p* = 0.26)^g^	−0.1484 (*p* = 0.61)^e^	−0.2618 (*p* < 0.05)^h^

^a^*n* = 12, ^b^ *n* = 54, ^c^ *n* =11, ^d^ *n* = 51, ^e^ *n* = 14, ^f^ *n* = 52, ^g^ *n* = 39 and ^h^ *n* = 48. *n* = 25 non-diabetic and 75 diabetic patients affected by MetS in all other cases. BMI: body mass index, HOMA: homeostasis model assessment of insulin resistance, TNF-α: tumor necrosis factor a, hs-CRP: high sensitive C reactive protein and GLP-1: glucose-like peptide-1.

**Table 3:** Plasma insulin/glucose ratio.

Subjects	Glycemia	Insulinemia	Insulin/Glucose Ratio
	(mM)	(U/mL)	(µU per ml/mM)
Non-obese control subjects (*n* = 20)	4.78 ± 0.11	11.05 ± 0.88	2.31 ± 0.17
Obese control subjects (*n* = 20)	5.73 ± 0.07	28.18 ± 1.08	4.91 ± 0.16
Male MetS patients (*n* = 60)	7.56 ± 0.15	30.97 ± 0.49	4.16 ± 0.08
Female MetS patients (*n* = 40)	7.42 ± 0.17	33.14 ± 0.54	4.53 ± 0.09
Non-diabetic MetS patients (*n* = 25)	6.02 ± 0.08	30.38 ± 0.37	5.19 ± 0.12
Diabetic MetS patients (*n* = 75)	8.00 ± 0.09	32.33 ± 0.45	4.05 ± 0.07
Overweight MetS patients (*n* = 20)	6.82 ± 0.23	28.27 ± 0.38	4.26 ± 0.17
Obese MetS patients (*n* = 80)	7.68 ± 0.12	32.73 ± 0.40	4.32 ± 0.12

**Table 4:** Correlation analyses between the insulinogenic index and selected variables in diabetic and non-diabetic MetS patients.

**BMI**		**C-Peptide**		**HOMA-IR Index**	
Non-diabetic	Diabetic	Non-diabetic	Diabetic	Non-diabetic	Diabetic
+0.0853 (*p* = 0.68)	+0.3856 (*p* < 0.01)	−0.3426 (*p* = 0.09)	+0.2859 (*p* < 0.01)	−0.1173 (*p* < 0.57)	+0.2699 (*p* < 0.01)
**Glycated hemoglobin (HbA1C)**		**Total cholesterol**		**HDL cholesterol**	
Non-diabetic	Diabetic	Non-diabetic	Diabetic	Non-diabetic	Diabetic
+0.5669 (*p* < 0.01)	+0.1765 (*p* = 0.12)	+0.2139 (*p* = 0.30)	+0.2250 (*p* < 0.05)	+0.1897 (*p* = 0.36)	+0.0866 (*p* = 0.46)
**LDL cholesterol**		**Triglycerides**		**Leptin**	
Non-diabetic	Diabetic	Non-diabetic	Diabetic	Non-diabetic	Diabetic
+0.1786 (*p* = 0.39)	+0.0626 (*p* = 0.59)	+0.0127 (*p* = 0.95)	+0.2155 (*p* < 0.05)	+0.0666 (*p* = 0.83)^a^	+0.3781 (*p* < 0.01)^b^
**Adiponectin**		**Interleukin-6**		**TNF-α**	
Non-diabetic	Diabetic	Non-diabetic	Diabetic	Non-diabetic	Diabetic
−0.141+ (*p* = 0.67)^c^	−0.0563 (*p* = 0.69)^d^	+0.2712 (*p* = 0.39)^a^	+0.2747 (*p* < 0.05)^d^	+0.5941 (*p* < 0.05)^f^	+0.0426 (*p* = 0.76)^e^
**hs-CRP**		**GLP-1**			
Non-diabetic	Diabetic	Non-diabetic	Diabetic		
+0.3484 (*p* = 0.29)^c^	+0.6560 (*p* < 0.01)^g^	+0.2304 (*p* = 0.42)^f^	−0.4838 (*p* < 0.01)^h^		

^a^*n* = 12, ^b^ *n* = 54, ^c^ *n* =11, ^d^ *n* = 51, ^e^ *n* = 52, ^f^ *n* =14, ^g^ *n* = 39 and ^h^ *n* = 48. *n* =25 non-diabetic and 75 diabetic patients affected by MetS in all other cases.

**Table 5:** Correlation analyses between HOMA-IR index and selected variables in male and female subjects.

Selected Variable	Sex	Correlation Index	*p*-Value
HbA1C HbA1C	Female	+0.8818	< 0.01
	Male	+0.7974	< 0.01
BMI	Male	+0.7077	< 0.01
BMI	Female	+0.7045	< 0.01
Leptin	Female	+0.6395	< 0.01
hs-CRP	Male	+0.5782	< 0.01
Interleukin-6	Male	+0.5431	< 0.01
Triglycerides	Male	+0.4850	< 0.01
Triglycerides	Female	+0.4342	< 0.01
hs-CRP	Female	+0.3989	= 0.08
TNF-α	Male	+0.2810	= 0.09
Interleukin-6	Female	+0.2199	= 0.27
Total cholesterol	Male	+0.1867	= 0.15
Total cholesterol	Female	−0.0205	= 0.90
LDL-cholesterol	Male	−0.0298	= 0.82
LDL-cholesterol	Female	−0.0620	= 0.70
GLP-1	Male	−0.1443	= 0.40
Leptin	Male	−0.2234	= 0.17
HDL-cholesterol	Female	−0.2448	= 0.13
Adiponectin	Female	−0.2552	= 0.22
TNF-α	Female	−0.2601	= 0.19
HDL-cholesterol	Male	−0.3262	= 0.01
Adiponectin	Male	−0.5088	< 0.01
GLP-1	Female	−0.7568	< 0.01

## References

[1] Aboul-Enein B.H., Bernstein J., Neary A.C. (2017). Dietary transition and obesity in selected Arabic-speaking countries: A review of the current evidence. East. Mediterr. Health J..

[2] Belahsen R. (2014). Nutrition transition and food sustainability. Proc. Nutr. Soc..

[3] Rahim H.F.A., Sibai A., Khader Y., Hwalla N., Fadhil I., Alsiyabi H., Mataria A., Mendis S., Mokdad A.H., Husseini A. (2014). Non-communicable diseases in the Arab world. Lancet.

[4] Diaf M., Khaled M.B. (2017). Overview on main nutrition-related diseases in three countries from North Africa. N. Afr. J. Food Nutr. Res.

[5] Toselli S., Gualdi-Russo E., Boulos D.N., Anwar W.A., Lakhoua C., Jaouadi I., Khyatti M., Hemminki K. (2014). Prevalence of overweight and obesity in adults from North Africa. Eur. J. Public Health.

[6] Lamri L., Gripiotis E., Ferrario A. (2014). Diabetes in Algeria and challenges for health policy: A literature review of prevalence, cost, management and outcomes of diabetes and its complications. Glob. Health.

[7] Melo B.F., Sacramento J.F., Ribeiro M.J., Prego C.S., Correia M.C., Coelho J.C., Cunha-Guimaraes J.P., Rodrigues T., Martins I.B., Guarino M.P. (2019). Evaluating the Impact of Different Hypercaloric Diets on Weight Gain, Insulin Resistance, Glucose Intolerance, and its Comorbidities in Rats. Nutrients.

[8] Julibert A., Bibiloni M.D.M., Bouzas C., Martínez-González M.Á., Salas-Salvadó J., Corella D., Zomeño M.D., Romaguera D., Vioque J., Alonso-Gómez Á.M. (2019). Total and Subtypes of Dietary Fat Intake and Its Association with Components of the Metabolic Syndrome in a Mediterranean Population at High Cardiovascular Risk. Nutrients.

[9] Vidal J., Jiménez A. (2016). Definition, History, and Management of the Metabolic Syndrome and Management Gaps. Metabolic Syndrome and Diabetes.

[10] Engin A. (2017). The definition and prevalence of obesity and metabolic syndrome. Obesity and Lipotoxicity.

[11] Robberecht H., Hermans N. (2016). Biomarkers of metabolic syndrome: Biochemical background and clinical significance. Metab. Syndr. Relat. Disord..

[12] Srikanthan K., Feyh A., Visweshwar H., Shapiro J.I., Sodhi K. (2016). Systematic review of metabolic syndrome biomarkers: A panel for early detection, management, and risk stratification in the West Virginian population. Int. J. Med Sci..

[13] Martin K.A., Mani M.V., Mani A. (2015). New targets to treat obesity and the metabolic syndrome. Eur. J. Pharmacol..

[14] Babikr W.G., Alshahrani A.S.A., Hamid H.G.M., Abdelraheem A.H.M.K., Shalayel M.H.F. (2016). The correlation of HbA1c with body mass index and HDL-cholesterol in type 2 diabetic patients. Biomed Res.

[15] Bae E., Cha R.-H., Kim Y.C., An J.N., Kim D.K., Yoo K.D., Lee S.M., Kim M.-H., Park J.T., Kang S.-W. (2017). Circulating TNF receptors predict cardiovascular disease in patients with chronic kidney disease. Medicine.

[16] Jung U.J., Choi M.-S. (2014). Obesity and its metabolic complications: The role of adipokines and the relationship between obesity, inflammation, insulin resistance, dyslipidemia and nonalcoholic fatty liver disease. Int. J. Mol. Sci..

[17] Danesh J., Wheeler J.G., Hirschfield G.M., Eda S., Eiriksdottir G., Rumley A., Lowe G.D., Pepys M.B., Gudnason V. (2004). C-reactive protein and other circulating markers of inflammation in the prediction of coronary heart disease. N. Engl. J. Med..

[18] Indulekha K., Surendar J., Anjana R.M., Geetha L., Gokulakrishnan K., Pradeepa R., Mohan V. (2015). Metabolic obesity, adipocytokines, and inflammatory markers in Asian Indians—CURES-124. Diabetes Technol. Ther..

[19] Lehr S., Hartwig S., Sell H. (2012). Adipokines: A treasure trove for the discovery of biomarkers for metabolic disorders. Proteom. Clin. Appl..

[20] de Abreu V.G., de Moraes Martins C.J., de Oliveira P.A.C., Francischetti E.A. (2017). High-molecular weight adiponectin/HOMA-IR ratio as a biomarker of metabolic syndrome in urban multiethnic Brazilian subjects. PLoS ONE.

[21] den Biggelaar L.J.C.J., Sep S.J.S., Eussen S.J.P.M., Mari A., Ferrannini E., van Greevenbroek M.M.J., van der Kallen C.J.H., Schalkwijk C.G., Stehouwer C.D.A., Dagnelie P.C. (2017). Discriminatory ability of simple OGTT-based beta cell function indices for prediction of prediabetes and type 2 diabetes: The CODAM study. Diabetologia.

[22] Woo Y.C., Cheung B.M.Y., Yeung C.Y., Lee C.H., Hui E.Y.L., Fong C.H.Y., Tso A.W.K., Tam S., Lam K.S.L. (2015). Cardiometabolic risk profile of participants with prediabetes diagnosed by HbA1c criteria in an urban Hong Kong Chinese population over 40 years of age. Diabet. Med..

[23] Drucker D.J. (2016). The cardiovascular biology of glucagon-like peptide-1. Cell Metab..

[24] Yamaoka-Tojo M., Tojo T., Takahira N., Matsunaga A., Aoyama N., Masuda T., Izumi T. (2010). Elevated circulating levels of an incretin hormone, glucagon-like peptide-1, are associated with metabolic components in high-risk patients with cardiovascular disease. Cardiovasc. Diabetol..

[25] Belhayara M.I., Mellouk Z., Hamdaoui M.S., Bachaoui M., Kheroua O., Malaisse W.J. (2019). Relationship between the insulin resistance and circulating predictive biochemical markers in metabolic syndrome among young adults in western Algeria. Diabetes Metab. Syndr. Clin. Res. Rev..

[26] (2002). National Cholesterol Education Program (NCEP) Expert Panel on Detection, Evaluation, and Treatment of High Blood Cholesterol in Adults (Adult Treatment Panel III). Third report of the National Cholesterol Education Program (NCEP) Expert Panel on detection, evaluation, and treatment of high blood cholesterol in adults (Adult Treatment Panel III). Circulation.

[27] Friedewald W.T., Levy R.I., Fredrickson D.S. (1972). Estimation of the concentration of low-density lipoprotein cholesterol in plasma, without use of the preparative ultracentrifuge. Clin. Chem..

[28] Matthews D.R., Hosker J.P., Rudenski A.S., Naylor B.A., Treacher D.F., Turner R.C. (1985). Homeostasis model assessment: Insulin resistance and β-cell function from fasting plasma glucose and insulin concentrations in man. Diabetologia.

[29] Mellouk Z., Zhang Y., Bulur N., Louchami K., Malaisse W.J., Ait Yahia D., Sener A. (2012). The metabolic syndrome of fructose-fed rats: Effects of long-chain polyunsaturated $ømega$3 and $ømega$6 fatty acids. III. Secretory behaviour of isolated pancreatic islets. Int. J. Mol. Med..

[30] Mellouk Z., Hachimi Idrissi T., Louchami K., Hupkens E., Malaisse W.J., Ait Yahia D., Sener A. (2011). The metabolic syndrome of fructose-fed rats: Effects of long-chain polyunsaturated $ømega$3 and $ømega$6 fatty acids. I. Intraperitoneal glucose tolerance test. Int. J. Mol. Med..

[31] Agardh C.-D., Ahrén B. (2012). Switching from high-fat to low-fat diet normalizes glucose metabolism and improves glucose-stimulated insulin secretion and insulin sensitivity but not body weight in C57BL/6J mice. Pancreas.

[32] Cancelas J., Prieto P.G., Villanueva-Peñacarrillo M.L., Valverde I., Malaisse W.J. (2006). Effects of an olive oil-enriched diet on glucagon-like peptide 1 release and intestinal content, plasma insulin concentration, glucose tolerance and pancreatic insulin content in an animal model of type 2 diabetes. Horm. Metab. Res..

[33] Chia C.W., Egan J.M., Ferrucci L. (2018). Age-related changes in glucose metabolism, hyperglycemia, and cardiovascular risk. Circ. Res..

[34] Laakso M., Kuusisto J. (2014). Insulin resistance and hyperglycaemia in cardiovascular disease development. Nat. Rev. Endocrinol..

[35] Malaisse W.J. (2014). Insulin release: The receptor hypothesis. Diabetologia.

[36] Fu Z., R Gilbert E., Liu D. (2013). Regulation of insulin synthesis and secretion and pancreatic Beta-cell dysfunction in diabetes. Curr. Diabetes Rev..

[37] Malin S.K., Finnegan S., Fealy C.E., Filion J., Rocco M.B., Kirwan J.P. (2014). β-Cell dysfunction is associated with metabolic syndrome severity in adults. Metab. Syndr. Relat. Disord..

[38] Cubeddu L.X., Hoffmann I.S. (2012). Impact of traits of metabolic syndrome on β-cell function and insulin resistance in normal fasting, normal glucose tolerant subjects. Metab. Syndr. Relat. Disord..

[39] Garg M.K., Dutta M.K., Mahalle N. (2011). Study of beta-cell function (by HOMA model) in metabolic syndrome. Indian J. Endocrinol. Metab..

[40] Baez-Duarte B.G., Sánchez-Guillén M.D.C., Perez-Fuentes R., Zamora-Ginez I., Leon-Chavez B.A., Revilla-Monsalve C., Islas-Andrade S. (2010). β-cell function is associated with metabolic syndrome in Mexican subjects. DiabetesMetab. Syndr. Obes. Targets Ther..

[41] Yoon H., Yoon Y.S., Kim S.G., Oh H.J., Choi C.W., Seong J.M., Park J. (2019). Relationship between metabolic syndrome and metabolic syndrome score with β-cell function by gender in non-diabetic Korean populations. Endocr. Res..

[42] Chen C., Cohrs C.M., Stertmann J., Bozsak R., Speier S. (2017). Human beta cell mass and function in diabetes: Recent advances in knowledge and technologies to understand disease pathogenesis. Mol. Metab..

[43] Henquin J.-C., Dufrane D., Kerr-Conte J., Nenquin M. (2015). Dynamics of glucose-induced insulin secretion in normal human islets. Am. J. Physiol. -Endocrinol. Metab..

[44] DeFronzo R.A., Abdul-Ghani M.A. (2011). Preservation of β-cell function: The key to diabetes prevention. J. Clin. Endocrinol. Metab..

[45] Meigs J.B., Wilson P.W., Fox C.S., Vasan R.S., Nathan D.M., Sullivan L.M., D’Agostino R.B. (2006). Body mass index, metabolic syndrome, and risk of type 2 diabetes or cardiovascular disease. J. Clin. Endocrinol. Metab..

[46] Vakilian M., Tahamtani Y., Ghaedi K. (2019). A review on insulin trafficking and exocytosis. Gene.

[47] Kojima I., Medina J., Nakagawa Y. (2017). Role of the glucose-sensing receptor in insulin secretion. Diabetes Obes. Metab..

[48] Malaisse W.J. (2016). Role of glycogen metabolism in pancreatic islet beta cell function. Diabetologia.

[49] Ashcroft F.M., Rohm M., Clark A., Brereton M.F. (2017). Is type 2 diabetes a glycogen storage disease of pancreatic β cells?. Cell Metab..

[50] Nagy L., Márton J., Vida A., Kis G., Bokor É., Kun S., Gönczi M., Docsa T., Tóth A., Antal M. (2018). Glycogen phosphorylase inhibition improves beta cell function. Br. J. Pharmacol..

[51] Malaisse W.J., Marynissen G., Sener A. (1992). Possible role of glycogen accumulation in B-cell glucotoxicity. Metabolism.

[52] Malaisse W.J. (1991). The anomeric malaise: A manifestation of B-cell glucotoxicity. Horm. Metab. Res..

[53] Owei I., Umekwe N., Provo C., Wan J., Dagogo-Jack S. (2017). Insulin-sensitive and insulin-resistant obese and non-obese phenotypes: Role in prediction of incident pre-diabetes in a longitudinal biracial cohort. BMJ Open Diabetes Res. Care.

[54] Iwani N.A.K.Z., Jalaludin M.Y., Zin R.M.W.M., Fuziah M.Z., Hong J.Y.H., Abqariyah Y., Mokhtar A.H., Nazaimoon W.M.W. (2017). Triglyceride to HDL-C ratio is associated with insulin resistance in overweight and obese children. Sci. Rep..

[55] Saravia G., Civeira F., Hurtado-Roca Y., Andres E., Leon M., Pocovi M., Ordovas J., Guallar E., Fernandez-Ortiz A., Casasnovas J.A. (2015). Glycated hemoglobin, fasting insulin and the metabolic syndrome in males. cross-sectional analyses of the Aragon Workers’ Health Study baseline. PLoS ONE.

[56] Farid S.M. (2018). Study of Correlation Between Anthropometric Parameters (BMI, WC, WHR) and Atherogenic Index of Plasma (AIP) in Type 2 Diabetics in Jeddah, Saudi Arabia. GJBB..

[57] Zadhoush F., Sadeghi M., Pourfarzam M. (2015). Biochemical changes in blood of type 2 diabetes with and without metabolic syndrome and their association with metabolic syndrome components. J. Res. Med Sci..

[58] Naveen L., Santoshi M., Madhav D., Sri Rama A.G., Mahesh V. (2014). A study of association of insulin resistance and cardio metabolic risk factors in an adult population with type 2 diabetes mellitus, Inter. J. Appl. Med. Sci..

[59] Raja Reddy R., Jayarama N., Shashidhar K.N. (2013). Association among HbA1c and lipid profile in Kolar type 2 diabetic population. J. Pharm. Sci. Innov..

[60] Bodhe C., Jankar D., Bhutada T., Patwardhan M., Patwardhan V. (2012). HbA1c: Predictor of dyslipidemia and atherogenicity in diabetes mellitus. Int. J. Basic Med Sci. Pharm..

[61] Bastenie P.A., Conard V., Franckson J., Bellens R., Malaisse W. (1963). Exploration des états prédiabétiques. Bull. De L’académie R. De Médecine De Belg..

